# A revision of the *Stenus flammeus* group (Coleoptera, Staphylinidae) with descriptions of twelve new species

**DOI:** 10.3897/zookeys.595.8752

**Published:** 2016-06-02

**Authors:** Liang Tang, Si-Yu Liu, Tong Niu

**Affiliations:** 1 Department of Biology, Shanghai Normal University, 100 Guilin Road, 1st Educational Building 323 Room, Shanghai, 200234 P. R. China

**Keywords:** China, Coleoptera, identification key, new species, Staphylinidae, Stenus flammeus group

## Abstract

The *Stenus flammeus* group is proposed and twelve new species of the group are described: *Stenus corniculus* **sp. n.**, *Stenus daicongchaoi* **sp. n.**, *Stenus jiajinshanus* **sp. n.**, *Stenus jindingianus* **sp. n.**, *Stenus paraflammeus* **sp. n.**, *Stenus pengzhongi* **sp. n.**, *Stenus pseudoflammeus* **sp. n.**, *Stenus punctidorsus* **sp. n.**, *Stenus tuyueyei* **sp. n.**, *Stenus xilingmontis* **sp. n.**, and *Stenus zhoudeyaoi* sp. n. from Sichuan Province, and *Stenus dabashanus* **sp. n.** from Shaanxi Province. Their diagnostic characters are illustrated and a key to species of the group is provided.

## Introduction

The *Stenus flammeus* complex consisting of *Stenus flammeus* Tang & Puthz, 2008 and *Stenus bostrychus* Tang & Puthz, 2008 (both see Tang, Zhao & Puthz, 2008) was established by Tang, Zhao & Puthz in 2008 and it was assigned to the *Stenus cirrus* group at that time. With more material collected and in-depth understanding of the Chinese fauna of the genus *Stenus* increasing, it is clarified now that *Stenus flammeus* and its affinis represent a separate species group which may not be closely related to the *Stenus cirrus* group. The distributional range of the *Stenus flammeus* group is known only from the Sichuan Basin, while the range of the *Stenus cirrus* group is much larger, covering the most central area of East Asia from Vietnam (Puthz 1981), Hainan (Puthz 2003) and Taiwan (Puthz 2009) to Shaanxi (Tang et al. 2005) and Japan (Naomi 2004). Although a unique character in the genus, bearing long suberect setae on the abdomen, is shared by both groups, the members of the *Stenus flammeus* group differ from those of the *Stenus cirrus* group by the following characters: paraglossa coniform, while they are oval in the *Stenus cirrus* group; male sternite IX with apicolateral projections relatively long and posterior margin usually with median projection (exceptions: *Stenus xilingmontis*, *Stenus dabashanus*, *Stenus jiajinshanus*, *Stenus zhoudeyaoi*), while in the *Stenus cirrus* group, male sternite IX with apicolateral projections relatively short and posterior margin almost without median projection; spermatheca weakly sclerotized or even difficult to be observed although basal porch and/or basal duct are usually strongly sclerotized, while in the *Stenus cirrus* group, the entire spermatheca is always sclerotized in mature females.

## Material and methods

The specimens examined in this paper were collected by sifting leaf litters in forests and killed with ethyl acetate. For examination of the genitalia, the last three abdominal segments were detached from the body after softening in hot water. The aedeagi or the spermathecae, together with other dissected pieces, were mounted in Euparal (Chroma Gesellschaft Schmidt, Koengen, Germany) on plastic slides. Photos of sexual characters were taken with a Canon G9 camera attached to an Olympus SZX 16 stereoscope; habitus photos were taken with a Canon macro photo lens MP-E 65 mm attached to a Canon EOS 7D camera and stacked with Zerene Stacker (http://www.zerenesystems.com/cms/stacker).

The type specimens treated in this study are deposited in the following public and private collections:

CNC
 Canadian National Collection of Insects, Ottawa, Ontario, Canada 


SHNU
 Department of Biology, Shanghai Normal University, P. R. China 


cPut
 private collection V. Puthz, Schlitz, Germany 


cSch
 private collection M. Schülke, Berlin, Germany 


cSme
 private collection A. Smetana, Ottawa, Canada 


cWat
 private collection Y. Watanabe, Tokyo, Japan 


MHNG
Muséum d’Histoire Naturelle, Genève, Switzerland 


The measurements of proportions are abbreviated as follows:

BL
 body length, measured from the anterior margin of the clypeus to the posterior margin of abdominal tergite X


FL
 forebody length, measured from the anterior margin of the clypeus to the apex of the elytra (apicolateral angle) 


HW
 width of head including eyes 


PW
 width of pronotum 


EW
 width of elytra 


PL
 length of pronotum 


EL
 length of elytra, measured from humeral angle 


SL
 length of elytral suture 


## Taxonomy

### Stenus flammeus

Taxon classificationAnimaliaColeopteraStaphylinidae

Tang & Puthz, 2008

Stenus flammeus Tang & Puthz, 2008 in Tang, Zhao & Puthz, 2008: 10.

#### Material examined.

**CHINA: Sichuan: Holotype**: ♂, Luding County, Hailuogou, alt. 2200–2300 m, 27.VII.2006, HU Jia-Yao & TANG Liang leg. (SHNU). **Paratypes**: 14♂♂21♀♀, same data as for the holotype (SHNU); 3♂♂8♀♀, same data but 28.VII.2006, HU Jia-Yao & TANG Liang leg. (SHNU)

#### Measurements.

BL: 4.0–5.7mm, FL: 2.0–2.1 mm. HW: 0.85–0.96 mm, PL: 0.68–0.77 mm, PW: 0.68–0.74 mm, EL: 0.72–0.80 mm, EW: 0.77–0.86 mm, SL: 0.49–0.54 mm. Head 1.05–1.11 times as wide as elytra, pronotum 0.99–1.03 times as long as wide, elytra 0.87–0.92 times as long as wide.

### Stenus bostrychus

Taxon classificationAnimaliaColeopteraStaphylinidae

Tang & Puthz, 2008

Stenus bostrychus Tang & Puthz, 2008 in Tang, Zhao & Puthz, 2008: 12.

#### Material examined.

**China: Sichuan: Holotype**: ♂, Tianquan County, Labahe Nature Reserve, alt. 2400–2600 m, 31.VII.2006, HU Jia-Yao & TANG Liang leg. (SHNU). **Paratypes**: 3♂♂5♀♀, same data as for the holotype (SHNU); 6♂♂ 2♀♀, Tianquan County, Labahe Nature Reserve, alt. 2000 m, 30.VII.2006, HU Jia-Yao and TANG Liang leg. (SHNU); ♂, Labahe Nature Reserve, Tianquan County, Sichuan Prov., alt. 1900 m, 29.VII.2006, HU Jia-Yao & TANG Liang leg. (SHNU)

#### Measurements.

BL: 4.0–4.5mm, FL: 2.1–2.3 mm. HW: 0.84–0.92 mm, PL: 0.68–0.74 mm, PW: 0.65–0.71 mm, EL: 0.75–0.80 mm, EW: 0.73–0.81 mm, SL: 0.51–0.54 mm. Head 1.10–1.16 times as wide as elytra, pronotum 1.03–1.07 times as long as wide, elytra 0.98–1.03 times as long as wide.

### Stenus pengzhongi sp. n.

Taxon classificationAnimaliaColeopteraStaphylinidae

http://zoobank.org/414DE45D-513C-415A-A901-3956206D7136

Figs 1 , 13–17

#### Type material.

**Holotype. China: Sichuan**: ♂, glued on a card with labels as follows: “China: Sichuan Prov., Emei Shan, Xixiangchi, 29°33'N, 103°20'E, alt. 2100–2300 m, 21.VII.2012, Peng, Dai & Yin leg.” “Holotype / *Stenus pengzhongi* / Tang, Liu & Niu” [red handwritten label] (SHNU). **Paratypes.** 1♂, same data as for the holotype (SHNU); 1♀, Mt. Emei, 17.VII.2003, Hu & Tang leg. (SHNU); 1♂3♀♀, Emei Shan, 29°33'39"N, 103°20'42"E, 1850m, 23.V.2011, sift04, V. Grebennikov (CNC).

#### Description.

Brachypterous; Head black, other body parts dark brown, each elytron with a large orange spot, which is about 3/5 as long as and 1/2 as broad as the respective elytron. Antennae yellowish brown, club infuscate, maxillary palpi and legs yellowish brown.

BL: 3.1–3.4mm, FL: 1.5–1.6 mm.

HW: 0.71–0.76 mm, PL: 0.54–0.58 mm, PW: 0.50–0.55 mm, EL: 0.53–0.58 mm, EW: 0.57–0.58 mm, SL: 0.40–0.42 mm.

Head 1.25–1.31 times as wide as elytra; interocular area with two deep longitudinal furrows, median portion convex, slightly extending beyond the level of inner eye margins; punctures round, mostly well delimited, slightly larger and sparser on median area than those near inner margins of eyes, diameter of large punctures about as wide as apical cross section of antennal segment II; interstices smooth, narrower than half to entire diameter of punctures except those along the midline of the convex median portion, which may be twice as wide as diameter of punctures. Paraglossa coniform.

Pronotum 1.05–1.08 times as long as wide; disk slightly uneven, with distinct median longitudinal furrow, which is about 3/5 as long as pronotum; punctures more or less confluent, slightly larger than those of head; interstices smooth, distinctly narrower than half the diameter of punctures except those at the actual middle of longitudinal furrow, which could be three times as wide as diameter of punctures.

Elytra 0.95–0.98 times as long as wide; disk relatively even; punctures more or less longitudinally confluent, slightly larger than those of pronotum; interstices smooth, distinctly narrower than half the diameter of punctures.

Legs with tarsomeres IV deeply bilobed.

Abdomen cylindrical; line-like paratergites present only in segment III, tergites and sternites totally fused in segment IV–VI, tergite VII without apical membranous fringe; punctures mostly round, becoming slightly smaller posteriad; interstices smooth, mostly narrower than diameter of punctures.

Male. Sternite VIII (Fig. 13) with semi-circular emargination at middle of posterior margin; sternite IX (Fig. 14) with very long apicolateral projections, posterior margin with distinct median projection. Aedeagus (Figs 16, 17) slender; median lobe with apical sclerotized area semicircle; expulsion clasps large, strongly sclerotized; parameres extending a little beyond apex of median lobe, swollen at apical parts, each with 8–10 setae on apico-internal margins.

**Figures 1–6.**
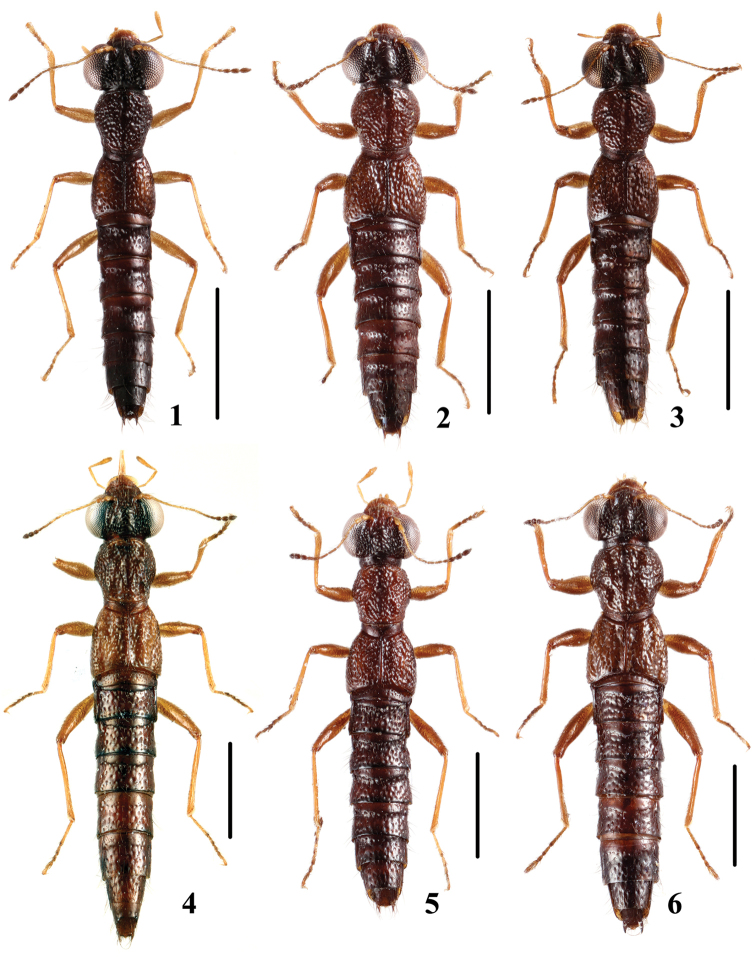
Habitus. **1** *Stenus pengzhongi* sp. n. **2** *Stenus jindingianus* sp. n. **3** *Stenus corniculus* sp. n. **4** *Stenus daicongchaoi* sp. n. **5** *Stenus punctidorsus* sp. n. **6** *Stenus paraflammeus* sp. n. Scale bars: 1 mm.

**Figures 7–12.**
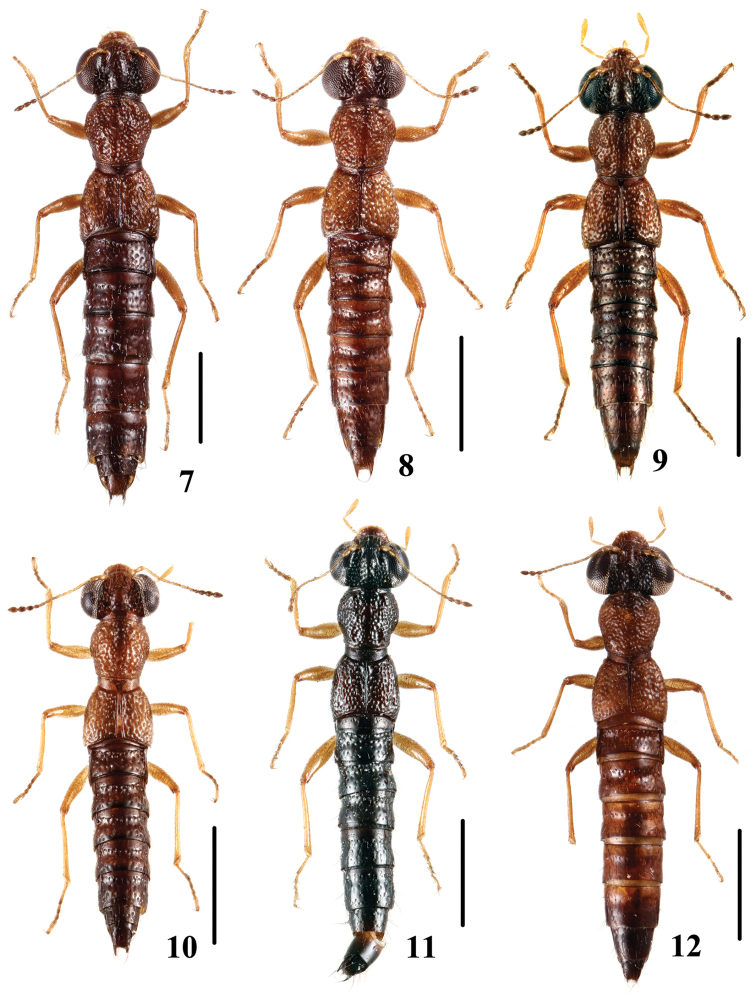
Habitus. **7** *Stenus pseudoflammeus* sp. n. **8** *Stenus xilingmontis* sp. n. **9** *Stenus zhoudeyaoi* sp. n. **10** *Stenus jiajinshanus* sp. n. **11** *Stenus tuyueyei* sp. n. **12** *Stenus dabashanus* sp. n. Scale bars: 1 mm.

**Figures 13–17.**
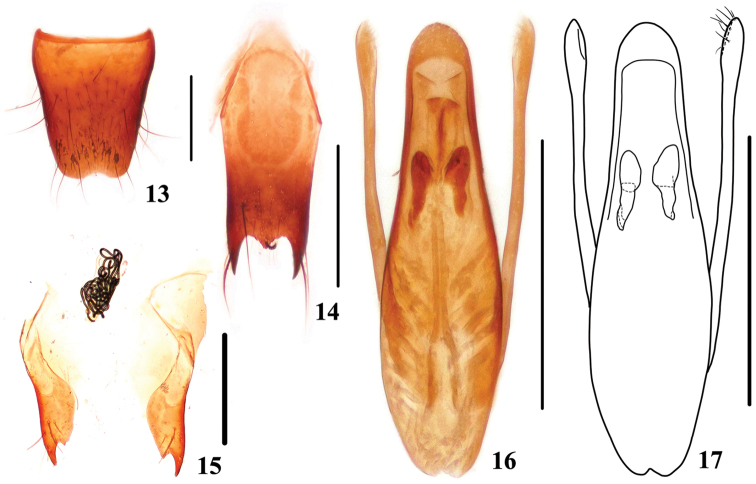
*Stenus pengzhongi*. **13** male sternite VIII **14** male sternite IX **15** valvifers and spermatheca **16, 17** aedeagus. Scale bars: 0.25 mm.

Female. Sternite VIII inconspicuously prominent at middle of posterior margin; sclerotized spermatheca (Fig. 15) with very complicated bends.

#### Distribution.

China (Sichuan).

#### Remarks.

The species can be distinguished from other related species by the following characters: body size smaller, surfaces of pronotum and elytra rather even and ratio of HW/EW larger.

#### Etymology.

This species is named in honor of Mr. Zhong Peng who collected some specimens of the new species.

### Stenus jindingianus sp. n.

Taxon classificationAnimaliaColeopteraStaphylinidae

http://zoobank.org/871D3DAF-8684-430D-8221-7331EF903116

Figs 2 , 18–23

#### Type material.

**Holotype. China: Sichuan**: ♂, glued on a card with labels as follows: “China: Sichuan Prov., Emeishan Mt., Jinding, 1.35 km, 29°31'N, 103°20'E, alt. 2800–3000 m, 19.VII.2012, Peng, Dai & Yin leg.” “Holotype / *Stenus jindingianus* / Tang, Liu & Niu” [red handwritten label] (SHNU). **Paratypes.** 1♂2♀♀, same data as for the holotype (SHNU); 1♂1♀, Emei Shan, 3000 m, 29°32'N, 103°21'E, 17.VII.1996, Smetana, Farkač & Kabátek leg. (cSme); 2♂♂, Emeishan, Jinding, 3020 m, 2.XI.1995, Uéno leg. (cWat); 2♂♂1♀, Emeishan, Taiziping, 2930 m, 2.XI.1995, Uéno leg. (cWat); 2♂♂, Emeishan, below Jinding, 3000 m, 5.X.1995, Nomura leg. (cWat).

#### Description.

Brachypterous; head broadly black along the inner eye margins, median portion dark brown or rarely black, labrum reddish brown, rest body parts reddish brown, elytra each with trace of yellow spot at humeral impression. Antennae, maxillary palpi and legs yellowish brown except antennal club infuscate.

BL: 3.1–3.3mm, FL: 1.6–1.7 mm.

HW: 0.73–0.76 mm, PL: 0.53–0.59 mm, PW: 0.58–0.62 mm, EL: 0.56–0.64 mm, EW: 0.68–0.71 mm, SL: 0.39–0.46 mm.

Head 1.03–1.07 times as wide as elytra, interocular area with two deep longitudinal furrows, median portion convex, slightly extending beyond the level of inner eye margins; punctures round, slightly confluent, slightly larger and sparser on median area than those near inner margins of eyes, diameter of large punctures about as wide as apical cross section of antennal segment II; interstices smooth, much narrower than half the diameter of punctures except those along the midline of the convex median portion, which may be twice as wide as diameter of punctures. Paraglossa coniform.

Pronotum 0.91–0.95 times as long as wide, disk uneven, with broad median longitudinal furrow throughout, two shallow impressions in anterior half, two shallow impressions in about middle, two shallow impressions in posterior half; punctures confluent, of similar size to those of head; interstices smooth, narrower than half the diameter of punctures except those at the bottom of longitudinal furrow, which could be much larger.

Elytra 0.82–0.90 times as long as wide; disk moderately even with indistinct longitudinal humeral impression, indistinct postero-lateral impression and indistinct long sutural impression, suture moderately convex; punctation strongly and longitudinally confluent; interstices smooth, very narrow and ridge-like.

Legs with tarsomeres IV deeply bilobed.

Abdomen cylindrical; paratergites very narrow and punctate, present only in segment III, tergites and sternites totally fused in segment IV–VI, tergite VII without apical membranous fringe; punctures gradually becoming smaller posteriad, punctures of abdominal tergites III slightly larger than those of pronotum in average; interstices smooth except sometimes traces of reticulation presented at last three tergites, narrower than the diameter of punctures on tergites III–VI.

**Figures 18–23.**
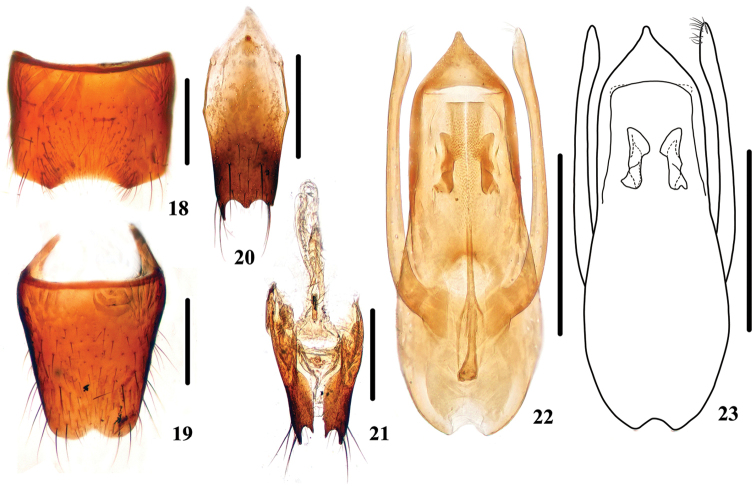
*Stenus jindingianus*. **18** male sternite VII **19** male sternite VIII **20** male sternite IX **21** valvifers and spermatheca **22, 23** aedeagus. Scale bars: 0.25 mm.

Male. Sternite VII (Fig. 18) with emargination at middle of posterior margin and a distinct impression before it; sternite VIII (Fig. 19) with semi-circular emargination at middle of posterior margin; sternite IX (Fig. 20) with very long apicolateral projections, posterior margin with distinct median projection. Aedeagus (Figs 21, 22) with apical sclerotized area triangular; expulsion clasps mediun in size; parameres as long as median lobe, slightly swollen at apical parts, each with 12–14 setae on apico-internal margins.

Female. Sternite VIII entire; spermatheca (Fig. 21) with basal duct strongly sclerotized, remining part of the spermathecal duct slightly sclerotized with multiple bends.

#### Distribution.

China (Sichuan).

#### Remarks.

The species can be distinguished from other related species except *Stenus corniculus* sp. n. by smaller body size and longitudinally confluent punctation of elytra. To distinguish from *Stenus corniculus* sp. n., see diagnoses of the latter.

#### Etymology.

The specific name is derived from the type locality of this species.

### Stenus corniculus sp. n.

Taxon classificationAnimaliaColeopteraStaphylinidae

http://zoobank.org/365E42E5-8C8D-4E9A-B099-C5B813DF4040

Figs 3 , 24–29

#### Type material.

**Holotype. China: Sichuan**: ♂, glued on a card with labels as follows: “China: Sichuan Prov., Emeishan Mt., Jieyin Palace, 0.7 km, 29°32'N, 103°20'E, alt. 2500–2600 m, 18.VII.2012, Peng, Dai & Yin Leg.” “Holotype / *Stenus corniculus* / Tang, Liu & Niu” [red handwritten label] (SHNU). **Paratypes.** 11♂♂9♀♀, same data as for the holotype (1 pair in cPut, rest in SHNU); 1♂2♀♀, Emei Shan, Leidongping, 2500 m, 29°32'N, 103°21'E, 18.VII.1996, Smetana, Farkač & Kabátek leg. (cSme); 1♂1♀, Emeishan, 29°32'37.3"N, 103°19'57.5"E, 2440 m, 18.VII.2010, sifting, V. Grebennikov (CNC); 1♀, Emeishan, 29°32'57.2"N, 103°20'37.7"E, 2289 m, 16.VII.2010, sifting, V. Grebennikov leg. (CNC); 1♀, Emeishan, Xixiangchi, 29.VII.2009, He & Tang leg. (SHNU)

#### Description.

BL: 3.4–3.7mm, FL: 1.6–1.8 mm.

HW: 0.71–0.81 mm, PL: 0.55–0.59 mm, PW: 0.55–0.61 mm, EL: 0.58–0.65 mm, EW: 0.61–0.71 mm, SL: 0.46–0.48 mm. Head 1.11–1.17 times as wide as elytra, pronotum 0.95–1.02 times as long as wide, elytra 0.91–0.96 times as long as wide.

Similar to *Stenus jindingianus* sp. n. in most aspects, but differs in the following characters: Head with interocular area entirely black; the traces of elytral marks more distinct and larger; abdominal tergites with punctures relatively sparser especially those of posterior area of each tergite.

**Figures 24–29.**
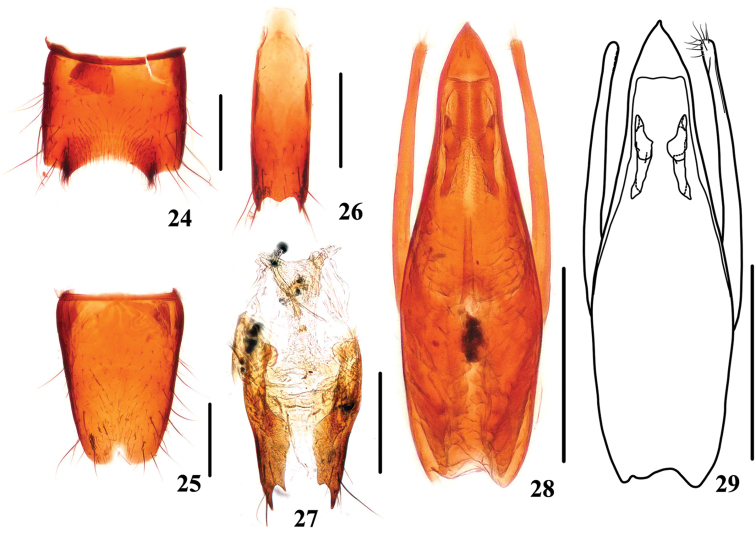
*Stenus corniculus*. **24** male sternite VII **25** male sternite VIII **26** male sternite IX **27** valvifers and spermatheca **28, 29** aedeagus. Scale bars: 0.25 mm.

Male. Sternite VII (Fig. 24) impressed at posteromedian part delimited by edged ridge on each side, posterior margin of the impression emarginated with a sharp projection on each side; sternite VIII (Fig. 25) with triangular emargination at middle of posterior margin; sternite IX (Fig. 26) with long apicolateral projections, posterior margin with broad and shallow median projection. Aedeagus (Figs 28, 29) slender; with median lobe broadest at about basal 2/5 and gradually tapering apicad, apical sclerotized area with an apical cuspidate projection; expulsion clasps large; parameres distinctly shorter than median lobe, slightly swollen at apical parts, each with 9–11 setae on apico-internal margins.

Female. Sternite VIII entire; sclerotized spermatheca (Fig. 27) consisting of short basal duct and long folded duct.

#### Distribution.

China (Sichuan).

#### Remarks.

The species resembles *Stenus jindingianus* sp. n. in most aspects, the main differences between them appear in proportions of pronotum and elytra.

#### Etymology.

The specific name is a combination of the Latin words “cornic” and “ulus” after its sternite VII decorated by sharp projections.

### Stenus daicongchaoi sp. n.

Taxon classificationAnimaliaColeopteraStaphylinidae

http://zoobank.org/248993F5-CC9B-44ED-8CB2-E47BB18F0897

Figs 4 , 30–35

#### Type material.

**Holotype. China: Sichuan**: ♂, glued on a card with labels as follows: “China: Sichuan Prov., Emeishan Mt., Jieyin Palace, 0.7 km, 29°32'N, 103°20'E, alt. 2500–2600 m, 18.VII.2012, Peng, Dai & Yin leg.”“Holotype / *Stenus daicongchaoi* / Tang Liu & Niu” [red handwritten label] (SHNU). **Paratypes.** 2♂♂2♀♀, same data as for the holotype (1 pair in cPut, rest in SHNU); 2♀♀, Emei Shan, Xixiangchi, 29°33'N, 103°20'E, alt. 2100–2300 m, 21.VII.2012, Peng, Dai & Yin leg. (SHNU); 1♂1♀, Emeishan Mt., Jinding, 29°31'N, 103°20'E, alt. 3000 m, 20.VII.2012, Peng, Dai & Yin leg. (SHNU); 1♀, Emeishan, 29°32'48.4"N, 103°20'06.3"E, 2342 m, 17.VII.2010, sifting, V. Grebennikov leg. (CNC);

#### Description.

Brachypterous; body brownish except head with interocular area somewhat darker and elytra lighter, each elytron with slender spot, which is about 1/2 as long as and 1/3 as broad as the respective elytron, sometimes they are inconspicuous. Antennae, maxillary palpi and legs yellowish brown except antennal club infuscate.

BL: 4.7–5.0mm, FL: 2.2–2.4 mm.

HW: 0.87–0.89 mm, PL: 0.70–0.74 mm, PW: 0.67–0.69 mm, EL: 0.79–0.82 mm, EW: 0.81–0.83 mm, SL: 0.58–0.62 mm.

Head 1.06–1.08 times as wide as elytra; interocular area with two deep longitudinal furrows, median portion convex, reaching the level of inner eye margins; punctures round, partly confluent, slightly larger and sparser on median area than those near inner margins of eyes, diameter of large punctures about as wide as apical cross section of antennal segment II; interstices smooth, much narrower than half the diameter of punctures except those along the midline of the convex median portion, which may be twice as wide as the diameter of punctures. Paraglossa coniform.

Pronotum 1.04–1.07 times as long as wide; disk uneven, with distinct median longitudinal furrow, two impressions in anterior half, transverse impression in the middle, and two impressions in posterior half; punctures more or less confluent, of similar size to those of head; interstices partially and indistinctly reticulated, more or less narrower than half the diameter of punctures except those at the bottom of longitudinal furrow, which could be larger.

Elytra 0.98–0.99 times as long as wide; disk uneven with longitudinal humeral impression, postero-lateral impression and deep sutural impression, suture moderately convex; punctation longitudinally confluent; interstices smooth and ridge-like.

Legs with tarsomeres IV deeply bilobed.

Abdomen cylindrical; paratergites very narrow and punctate, present only in segment III, tergites and sternites totally fused in segment IV–VI, tergite VII without apical membranous fringe; punctures round, becoming slightly smaller posteriad; interstices smooth except those of last two tergites shallowly reticulated, narrower than half the diameter of punctures on tergite III–VI.

**Figures 30–35.**
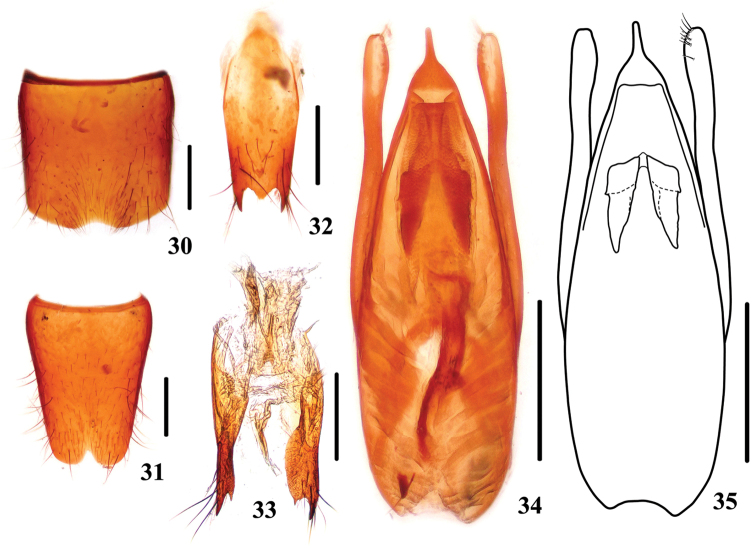
*Stenus daicongchaoi*. **30** male sternite VII **31** male sternite VIII **32** male sternite IX **33** valvifers and spermatheca **34, 35** aedeagus. Scale bars: 0.25 mm.

Male. Sternite VII (Fig. 30) impressed at posteromedian part with emargination along posterior margin of impression; sternite VIII (Fig. 31) with triangular emargination at middle of posterior margin; sternite IX (Fig. 32) with very long apicolateral projections, posterior margin with distinct median projection. Aedeagus (Figs 34, 35) broadest at about basal 2/5 and gradually tapering apicad, apical sclerotized area with a long apical cuspidate projection; expulsion clasps large; parameres a little shorter than median lobe, swollen at apical parts, each with 9–11 setae on apico-internal margins.

Female. Sternite VIII entire; spermatheca (Fig. 33) weekly sclerotized except the basal porch and basal duct well sclerotized.

#### Distribution.

China (Sichuan).

#### Remarks.

The species is similar to *Stenus bostrychus*, but can be distinguished from the latter by less confluent punctation of pronotum and relatively larger punctures of abdomen.

#### Etymology.

This species is named in honor of Mr. Cong-Chao Dai who collected some specimens of the new species.

### Stenus punctidorsus sp. n.

Taxon classificationAnimaliaColeopteraStaphylinidae

http://zoobank.org/01952264-6685-4622-8F1B-003F4D343F5E

Figs 5 , 36–40

#### Type material.

**Holotype. China: Sichuan**: ♂, glued on a card with labels as follows: “China: Sichuan Prov., Erlangshan Mt., 29°32'N, 102°18'E, alt. 2800–3000 m, 13.VII.2012, Peng, Dai & Yin leg.” “Holotype / *Stenus punctidorsus* / Tang, Liu & Niu” [red handwritten label] (SHNU). **Paratypes.** 1♂, Ya’an Pref., Tianquan Co., E Erlang Shan Pass, 2900 m, 9 km SE Luding, 29°52'N, 102°18'E, Gesiebe, 22.Vi.1999, leg. M. Schülke (cSch).

#### Description.

Brachypterous; Body reddish brown except head with interocular area blackish, each elytron with a very vague and inconspicuous small spot. Antennae, maxillary palpi and legs yellowish brown except antennal club infuscate.

BL: 3.4–3.9 mm, FL: 1.8–1.9 mm.

HW: 0.73–0.83 mm, PL: 0.58–0.63 mm, PW: 0.57–0.60 mm, EL: 0.61–0.67 mm, EW: 0.65–0.73 mm, SL: 0.45–0.50 mm.

Head 1.13–1.15 times as wide as elytra, interocular area with two deep longitudinal furrows, median portion convex, slightly extending beyond the level of inner eye margins; punctures round, more or less confluent, almost the same size, diameter of punctures about as wide as apical cross section of antennal segment II; interstices smooth, much narrower than half the diameter of punctures except those along the midline of the convex median portion, which may be slightly narrower than diameter of punctures. Paraglossa coniform.

Pronotum 1.02–1.05 times as long as wide; disk uneven, with broad median longitudinal furrow throughout, two impressions in anterior half each with an small outer tubercle, two impressions in about middle each with an inner tubercle, two deep impressions in posterior half each with an larger outer tubercle; punctures confluent, slightly smaller than those of head; interstices smooth, narrower than half the diameter of punctures except those at the bottom of longitudinal furrow, which could be much larger.

Elytra 0.92–0.94 times as long as wide; disk moderately uneven with distinct longitudinal humeral impression, distinct postero-lateral impression and long sutural impression, suture moderately convex; punctation and interstices similar to those of pronotum, except punctures slightly larger.

Legs with tarsomeres IV deeply bilobed.

Abdomen cylindrical; paratergites very narrow and punctate, present only in segment III, tergites and sternites totally fused in segment IV–VI, tergite VII without apical membranous fringe; punctures round, becoming slightly smaller posteriad; interstices smooth, narrower than half the diameter of punctures on tergite III–VI.

**Figures 36–40.**
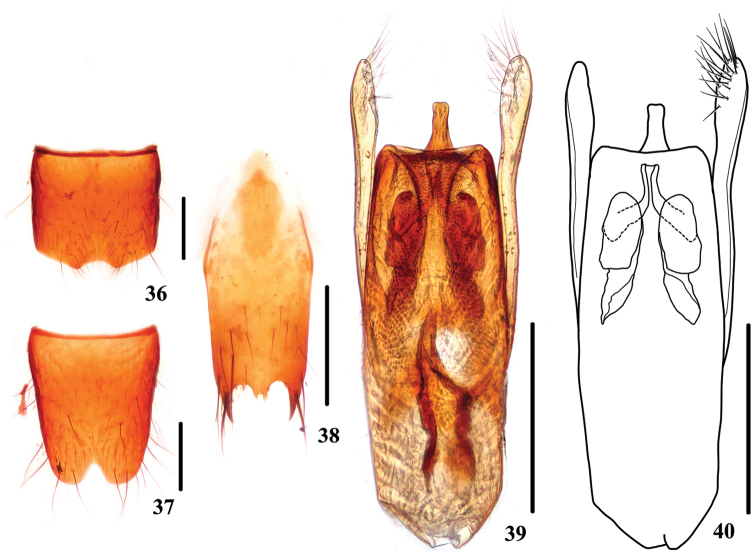
*Stenus punctidorsus*. **36** male sternite VII **37** male sternite VIII **38** male sternite IX **39, 40** aedeagus. Scale bars: 0.25 mm.

Male. Sternite VII (Fig. 36) impressed at posteromedian part with emargination along posterior margin of impression; sternite VIII (Fig. 37) with triangular emargination at middle of posterior margin; sternite IX (Fig. 38) with very long apicolateral projections, posterior margin with distinct median projection. Aedeagus (Figs 39, 40) with median lobe paralleled on sides, apical sclerotized area with a narrow and long apical projection; expulsion clasps large; parameres distinctly longer than median lobe, swollen at apical parts, each with 19–22 setae on apico-internal margins.

Female. Unknown.

#### Distribution.

China (Sichuan).

#### Remarks.

The new species is characterized by large and dense punctation of entire body especially of abdominal tergites.

#### Etymology.

The specific name is derived from the dense punctation of abdominal tergites.

### Stenus paraflammeus sp. n.

Taxon classificationAnimaliaColeopteraStaphylinidae

http://zoobank.org/6FE97A9D-A8B8-4B71-805F-172EEE90EF04

Figs 6 , 41–46

#### Type material.

**Holotype. China: Sichuan**: ♂, glued on a card with labels as follows: “China: Sichuan Prov., Tianquan County, Erlangshan Mt., Yakou 3.6km, 29°31'N, 102°17'E, alt. 2600–2800 m, 11.VII.2012, Peng, Dai & Yin leg.” “Holotype / *Stenus paraflammeus* / Tang Liu & Niu” [red handwritten label] (SHNU). **Paratypes.** 5 ♂♂4♀♀, same data as for the holotype (1pair in cPut, rest in SHNU); 1♂4♀♀, same data but 29°33'N, 102°18'E, alt. 2800–3000 m, 13.VII.2012, Peng, Dai & Yin leg. (SHNU); 3♂♂1♀, same data but 29°32'N, 102°18'E, alt. 2200–2300 m, 13.VII.2012, Peng, Dai & Yin leg. (SHNU); 1♂1♀, Erlangshan, 1600–2000 m, 29.VII.2006, Hu & Tang leg. (SHNU)

#### Description.

Brachypterous, reddish brown, head especially interocular area along the inner margins of eyes darker, abdomen moderately glossy. Antennae yellowish, club infuscate. Maxillary palpi yellowish. Legs reddish yellow, tarsomeres slightly infuscate.

BL: 4.4–4.7mm, FL: 2.0–2.3 mm.

HW: 0.90–1.00 mm, PL: 0.65–0.82 mm, PW: 0.72–0.85 mm, EL: 0.65–0.83 mm, EW: 0.83–0.96 mm, SL: 0.45–0.58 mm.

Head 1.06–1.08 times as wide as elytra; interocular area with two deep longitudinal furrows, median portion strongly convex, distinctly extending beyond the level of inner eye margins; punctures round, well delimited on the posterior areas of furrows and distinctly confluent on the rest parts especially on median portion, diameter of large punctures about as wide as medial cross section of 2nd antennal segment; interstices between punctures smooth, distinctly narrower than half the diameter of punctures. Paraglossa coniform.

Pronotum 0.90–0.96 times as long as wide; disk conspicuously uneven, with broad and deep median longitudinal furrow which begins from the anterior margin and ends at about basal 1/5, two deep impressions in anterior half each with an outer tubercle, two distinct impressions in about middle each with an inner tubercle, two deep impressions in posterior half each with an outer tubercle; punctures round and strongly confluent, mostly slightly smaller than those on frons; interstices smooth, much narrower than half the diameter of punctures except in median furrow, which is partially reticulated and broadly impunctate.

Elytra 0.85–0.89 times as long as wide; disk uneven with deep humeral impression, distinct postero-lateral impression and deep sutural impression, median portion between humeral impression and sutural impression distinctly convex; punctation irregular, punctures confluent, slightly larger than those on pronotum; interstices smooth, much narrower than half the diameter of punctures.

Legs with tarsomeres IV strongly bilobed.

Abdomen cylindrical; paratergites very narrow and punctate, present only in segment III, tergites and sternites totally fused in segment IV–VI, tergite VII without apical membranous fringe; punctures mostly round, becoming slightly smaller posteriad; interstices smooth on the basal four tergites and more or less sculptured on the rest tergites, narrower than half the diameter of punctures on basal three tergites and narrower than diameter of punctures on the following two tergites.

**Figures 41–46.**
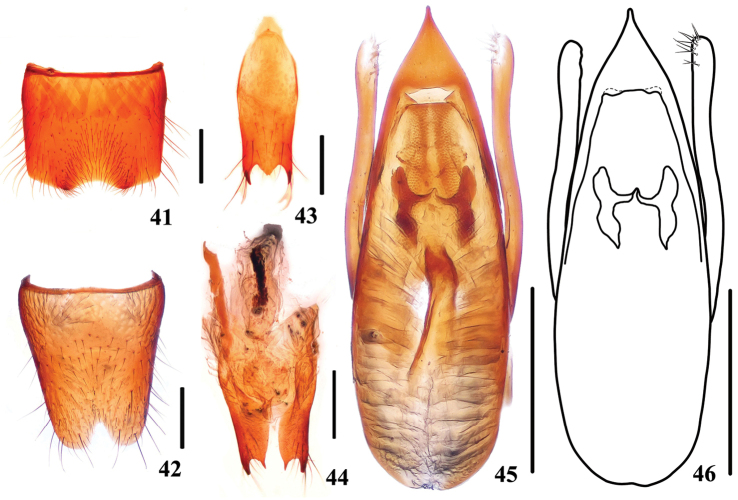
*Stenus paraflammeus*. **41** male sternite VII **42** male sternite VIII **43** male sternite IX **44** valvifers **45, 46** aedeagus. Scale bars: 0.25 mm.

Male. Seventh sternite (Fig. 41) deeply impressed at posteromedian part with emargination along posterior margin of impression; sternite VIII (Fig. 42) with triangular emargination at middle of posterior margin; sternite IX (Fig. 43) with very long apicolateral projections, posterior margin with strong median projection. Aedeagus (Figs 45, 46) with median lobe broadest at about basal 1/5 and gradually tapering apicad, apical sclerotized area with an apical cuspidate projection; expulsion clasps large; parameres distinctly shorter than median lobe, slightly swollen at apical parts, each with 10–12 setae on apico-internal margins.

Female. sternite VIII entire; valvifers (Fig. 44) each with very long inner tooth on posterior margin, spermatheca undetected.

#### Distribution.

China (Sichuan).

#### Remarks.

This new species is closely related to *Stenus flammeus* and *Stenus pseudoflammeus*, but can be easily distinguished from the latter two species by its broader pronotum.

#### Etymology.

The specific name is derived from the similar appearance of *Stenus flammeus*.

### Stenus pseudoflammeus sp. n.

Taxon classificationAnimaliaColeopteraStaphylinidae

http://zoobank.org/FB84F191-34E1-4231-AED9-A65BE9FC9E31

Figs 7 , 47–52

#### Type material.

**Holotype. China: Sichuan**: ♂, glued on a card with labels as follows: “China: Sichaun Prov., Dayi County, Xiling Xueshan, 30°41'59"N, 103°12'10"E, mixed leaf litter, shifted, 2150 m, 29.VII.2015, Jiang, Peng, Tu & Zhou leg.” “Holotype / *Stenus pseudoflammeus* / Tang, Liu & Niu” [red handwritten label] (SHNU). **Paratypes.** 3♂♂4♀♀, same data as for the holotype (1 pair in cPut, rest in SHNU); 1♂2♀♀, Xiling Mt., Dali, 1600-2400 m, 30.VII–4.VIII.1996, Kurbatov leg. (MHNG).

#### Description.

BL: 4.2–5.1mm, FL: 2.2–2.3 mm.

HW: 0.95–0.98 mm, PL: 0.74–0.78 mm, PW: 0.73–0.76 mm, EL: 0.83–0.87 mm, EW: 0.91–0.95 mm, SL: 0.52–0.54 mm. Head 1.03–1.06 times as wide as elytra, pronotum 1.01–1.03 times as long as wide, elytra 0.84–0.89 times as long as wide.

Similar to *Stenus paraflammeus* sp. n. in most aspects, but differs in the following characters: the convex median portion of head extending to the same level of inner eye margins; punctures of forebody relatively smaller, larger punctures on head about as wide as basal cross section of 2nd antennal segment; impressions of pronotum and elytra shallower; punctation of abdominal tergites slightly finer and sparser.

**Figures 47–52.**
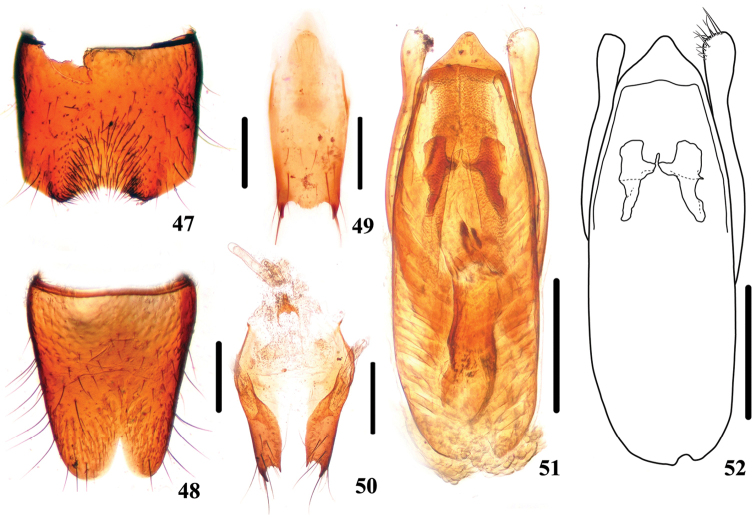
*Stenus pseudoflammeus*. **47** male sternite VII **48** male sternite VIII **49** male sternite IX **50** valvifers and spermatheca **51, 52** aedeagus. Scale bars: 0.25 mm.

Male. Sternites VII (Fig. 47) and VIII (Fig. 48) similar to those of *Stenus paraflammeus*; sternite IX (Fig. 49) with very long apicolateral projections, posterior margin with short broad serrate median projection. Aedeagus (Figs 51, 52) with median lobe robust, apical sclerotized area triangular; expulsion clasps large; parameres as long as median lobe, distinctly swollen at apex, each with 13–15 setae on apico-internal margins.

Female. Sternite VIII entire; spermatheca (Fig. 50) with basal porch strongly sclerotized, spermathecal duct slightly sclerotized and bent twice.

#### Distribution.

China (Sichuan).

#### Remarks.

The species is similar to *Stenus paraflammeus* but can be distinguished from the latter by slender pronotum; and it is also very similar to *Stenus flammeus*, dissections are necessary to distinguish them.

#### Etymology.

The specific name is derived from the similar appearance of *Stenus flammeus*.

### Stenus xilingmontis sp. n.

Taxon classificationAnimaliaColeopteraStaphylinidae

http://zoobank.org/F06D09EA-66D2-4C6A-8580-E95581B306B4

Figs 8 , 53–57

#### Type material.

**Holotype. China: Sichuan**: ♂, glued on a card with labels as follows: “China: Sichuan, Xiling Mt., 1300 m, litter, 30.VII.1996, S. Kurbatov leg.” “Holotype / *Stenus xilingmontis* / Tang, Liu & Niu” [red handwritten label] (MHNG). **Paratypes.** 2♂♂8♀♀, same data as for the holotype (1pair in SHNU, rest in MHNG).

#### Description.

Brachypterous; body reddish brown except head broadly blackish along the inner eye margins. Antennae, maxillary palpi and legs yellowish brown except antennal club infuscate.

BL: 3.1–3.7mm, FL: 1.6–1.8 mm.

HW: 0.70–0. 79 mm, PL: 0.53–0.63 mm, PW: 0.55–0.63 mm, EL: 0.58–0.67 mm, EW: 0.65–0.73 mm, SL: 0.47–0.55 mm.

Head 1.06–1.09 times as wide as elytra; interocular area with two deep longitudinal furrows, median portion convex, slightly extending beyond the level of inner eye margins; punctures round, slightly larger and sparser on median area than those near inner margins of eyes, diameter of large punctures about as wide as apical cross section of antennal segment II; interstices smooth, much narrower than half the diameter of punctures except those along the midline of the convex median portion, which may be 1.5 times as wide as diameter of punctures. Paraglossa coniform.

Pronotum 0.95–1.00 times as long as wide; disk uneven, with distinct median longitudinal furrow, two impressions in anterior half, transverse impression in the middle, and two impressions in posterior half; punctures partially confluent, of similar size to those of head; interstices smooth except few reticulations at the bottom of median furrow, more or less narrower than half the diameter of punctures except those at the bottom of longitudinal furrow, which may be twice as wide as diameter of punctures.

Elytra 0.89–0.93 times as long as wide; disk moderately uneven with distinct longitudinal humeral impression, distinct postero-lateral impression and long sutural impression, suture moderately convex; punctation and interstices similar to those of pronotum except punctures slightly confluent longitudinally.

Legs with tarsomeres IV deeply bilobed.

Abdomen cylindrical; paratergites very narrow and punctate, present only in segment III, tergites and sternites totally fused in segment IV–VI, tergite VII without apical membranous fringe; punctures of abdominal tergites III–VIII round, gradually becoming smaller posteriad; interstices smooth except those of last three tergites more or less shallowly reticulated, narrower than half the diameter of to the diameter of punctures.

**Figures 53–57.**
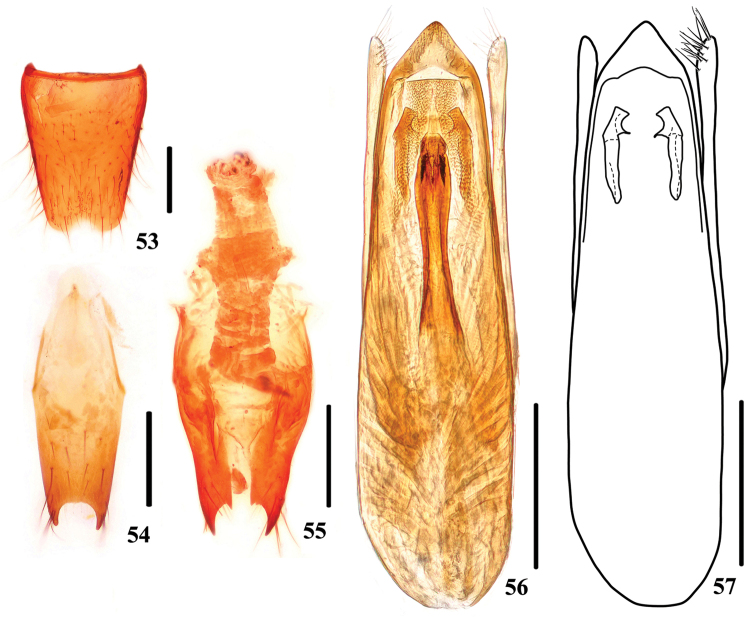
*Stenus xilingmontis*. **53** male sternite VIII **54** male sternite IX **55** valvifers **56, 57** aedeagus. Scale bars: 0.25 mm.

Male. Sternite VIII (Fig. 53) with semi-circular emargination at middle of posterior margin; sternite IX (Fig. 54) with very long apicolateral projections. Aedeagus (Figs 56, 57) slender, apical sclerotized area triangular; expulsion clasps large; parameres shorter than median lobe, slightly swollen at apex, each with 12–14 setae on apico-internal margins.

Female. Sternite VIII inconspicuously prominent at middle of posterior margin; valvifers as in fig. 55, sclerotized spermatheca undetected.

#### Distribution.

China (Sichuan).

#### Remarks.

The new species shares the same appearance with *Stenus zhoudeyaoi* sp. n., and both of them distributed on the same mountain. Dissections are necessary to distinguish them, though the altitude information will be also helpful.

#### Etymology.

The specific name is derived from the type locality of this species.

### Stenus zhoudeyaoi sp. n.

Taxon classificationAnimaliaColeopteraStaphylinidae

http://zoobank.org/4A56E419-E81B-48E0-9AB3-689C3340E5DF

Figs 9 , 58–62

#### Type material.

**Holotype. China: Sichuan**: ♂, glued on a card with labels as follows: “China: Sichaun Prov., Dayi County, Xiling Xueshan, 30°41'57"N, 103°09'44"E, mixed leaf litter, shifted, 3150 m, 28.VII.2015, Jiang, Peng, Tu & Zhou leg.” “Holotype / *Stenus zhoudeyaoi* / Tang, Liu & Niu” [red handwritten label] (SHNU). **Paratypes.** 2♂♂3♀♀, same data as for the holotype (1 pair in cPut, rest in SHNU).

#### Description.

BL: 3.3–3.6mm, FL: 1.6–1.7 mm.

HW: 0.67–0.77 mm, PL: 0.53–0.58 mm, PW: 0.54–0.60 mm, EL: 0.56–0.62 mm, EW: 0.64–0.71 mm, SL: 0.41–0.45 mm. Head 1.04–1.08 times as wide as elytra, pronotum 0.97–1.00 times as long as wide, elytra 0.87–0.91 times as long as wide.

Similar to *Stenus xilingmontis* sp. n. in most aspects, but differs in the following characters: Body coloration darker; punctation of pronotum and elytra less confluent. Sexual characters are also similar to *Stenus xilingmontis* sp. n. except the aedeagus and spermatheca. Aedeagus (Figs 61, 62) with apical sclerotized area larger and expulsion clasps smaller; parameres each with 9 or 10 setae on apico-internal margins. Spermatheca (Fig. 60) with very small sclerotized basal porch and basal duct, remining part of the duct very weakly sclerotized.

**Figures 58–62.**
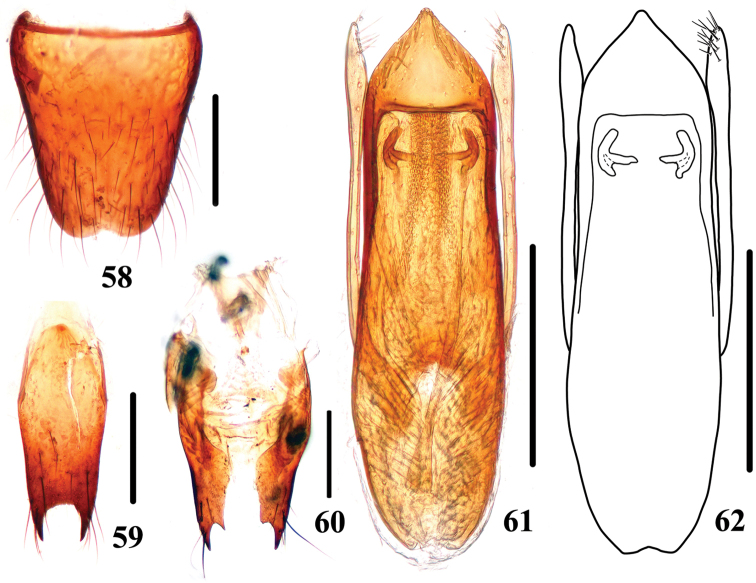
*Stenus zhoudeyaoi*. **58** male sternite VIII **59** male sternite IX **60** valvifers **61, 62** aedeagus. Scale bars: 0.25 mm.

#### Distribution.

China (Sichuan).

#### Remarks.

See that of *Stenus xilingmontis* sp. n.

#### Etymology.

This species is named in honor of Mr. De-Yao Zhou who collected some specimens of the new species.

### Stenus jiajinshanus sp. n.

Taxon classificationAnimaliaColeopteraStaphylinidae

http://zoobank.org/45EF8C89-BC0B-429C-91AB-D8D5C28DB424

Figs 10 , 63–67

#### Type material.

**Holotype. China: Sichuan**: ♂, glued on a card with labels as follows: “China: Sichuan Prov., Xiaojin County, Jiajin Shan, 30°48'49"N, 102°42'55"E, mixed leaf litter, sifted, 2490 m, 20.VII.2015, Jiang, Peng, Tu & Zhou leg.” “Holotype / *Stenus jiajinshanus* / Tang, Liu & Niu” [red handwritten label] (SHNU). **Paratypes.** 1♂, same data as for the holotype (SHNU); 1 ♀, Xiaojin County, Jiajin Shan, Mahuanggou, 30°51'20"N, 102°45'49"E, 2630 m, 21.VII.2015, Jiang, Peng, Tu & Zhou leg. (SHNU)

#### Description.

Brachypterous; forebody yellowish brown except areas along the inner margins of eyes slightly darker, abdomen reddish brown. Antennae, maxillary palpi and legs reddish yellow, except antennal club infuscate.

BL: 3.1–3.2mm, FL: 1.5–1.6 mm.

HW: 0.65–0.68 mm, PL: 0.48–0.49 mm, PW: 0.49–0.50 mm, EL: 0.47–0.50 mm, EW: 0.55–0.59 mm, SL: 0.35–0.40 mm.

Head 1.15–1.19 times as wide as elytra, interocular area with two deep longitudinal furrows, median portion convex, distinctly extending beyond the level of inner eye margins; punctures round, mostly well delimited, slightly larger and sparser on median area than those near inner margins of eyes, diameter of large punctures about as wide as apical cross section of antennal segment II; interstices smooth, much narrower than half the diameter of punctures except those along the midline of the convex median portion, which may be as wide as diameter of punctures. Paraglossa coniform.

Pronotum 0.98 times as long as wide, disk uneven, with distinct median longitudinal furrow, two impressions in anterior half, transverse impression in the middle, and two impressions in posterior half; punctures slightly confluent, of similar size to those of head; interstices smooth, more or less narrower than half the diameter of punctures except those at the bottom of longitudinal furrow, which could be larger.

Elytra 0.85–0.86 times as long as wide; disk moderately uneven with shallow longitudinal humeral impression, distinct postero-lateral impression and long sutural impression, suture moderately convex; punctures more or less longitudinally confluent, slightly larger than those of pronotum; interstices smooth, distinctly narrower than half the diameter of punctures.

Legs with tarsomeres IV deeply bilobed.

Abdomen cylindrical; line-like paratergites present only in segment III, tergites and sternites totally fused in segment IV–VI, tergite VII without apical membranous fringe; punctures of abdominal tergites III–VIII round to elliptic, gradually becoming smaller posteriad; interstices smooth except those of last two tergites shallowly reticulated, narrower than half the diameter of punctures.

**Figures 63–67.**
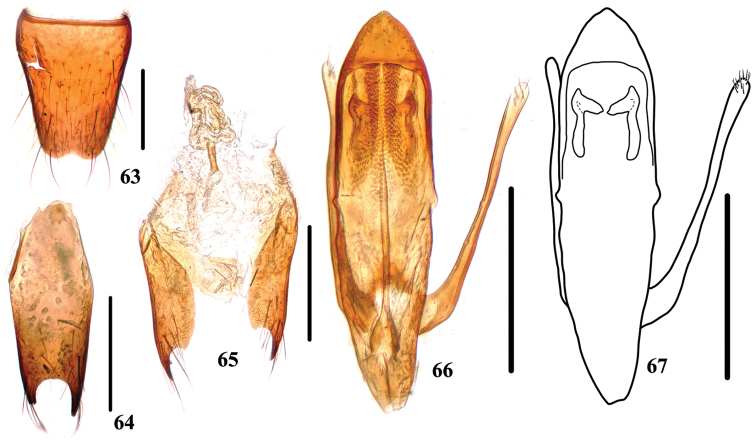
*Stenus jiajinshanus*. **63** male sternite VIII **64** male sternite IX **65** valvifers and spermatheca **66, 67** aedeagus. Scale bars: 0.25 mm.

Male. Sternite VIII (Fig. 63) with semi-circular emargination at middle of posterior margin; sternite IX (Fig. 64) with very long apicolateral projections. Aedeagus (Figs 66, 67) slender, apical sclerotized area subtriangular with round tip; expulsion clasps large; parameres distinctly shorter than median lobe, slightly swollen in apical part, with 9–10 setae on apico-internal margins.

Female. Sternite VIII entire; spermatheca (Fig. 65) with basal duct strongly sclerotized, remining part of the duct weakly sclerotized and very coiled.

#### Distribution.

China (Sichuan).

#### Remarks.

To distinguish the new species from other species with small body length, see characters listed in key.

#### Etymology.

The specific name is derived from the type locality of this species.

### Stenus tuyueyei sp. n.

Taxon classificationAnimaliaColeopteraStaphylinidae

http://zoobank.org/18B37947-DD90-4038-A558-8E1AD685C6AC

Figs 11 , 68–71

#### Type material.

**Holotype. China: Sichuan**: ♂, glued on a card with labels as follows: “China: Sichuan, Mianning Co., Yele, Daba, 26°55'22"N, 102°13'32"E, mixed leaf litter, sifted, 2500 m, 25.VI.2015, Jiang, Peng, Tu & Zhou leg.” “Holotype / *Stenus tuyueyei* / Tang, Liu & Niu” [red handwritten label] (SHNU).

#### Description.

Brachypterous; body blackish with pronotum and elytra somewhat lighter, antennae, maxillary palpi and legs reddish yellow, except antennal club infuscate.

BL: 3.8mm, FL: 1.8 mm.

HW: 0.73 mm, PL: 0.57 mm, PW: 0.57 mm, EL: 0.61 mm, EW: 0.64 mm, SL: 0.44 mm.

Head 1.14 times as wide as elytra; interocular area with two deep longitudinal furrows, median portion convex, reaching the level of inner eye margins; punctures round, slightly larger on posterior areas of furrows than those on rest areas, diameter of large punctures about as wide as basal cross section of antennal segment II; interstices smooth, much narrower than half the diameter of punctures except those along the midline of the convex median portion, which may be as wide as diameter of punctures. Paraglossa coniform.

Pronotum as long as wide; disk uneven, with distinct median longitudinal furrow, two impressions in anterior half, transverse impression in the middle, and two impressions in posterior half; punctures more or less confluent, of similar size to those of head; interstices faintly reticulated, more or less narrower than half the diameter of punctures except those at the bottom of longitudinal furrow, which could be three times as wide as diameter of punctures.

Elytra 0.95 times as long as wide; disk relatively uneven with shallow longitudinal humeral impression, distinct postero-lateral impression and long sutural impression, suture moderately convex; punctation and interstices similar to those of pronotum.

Legs with tarsomeres IV deeply bilobed.

Abdomen cylindrical; distinct paratergites absent, rudimentary lateral border present only on anterior half of segment III, tergites and sternites totally fused in posterior half of segment III and entire segment IV–VI; posterior margin of tergite VII without membranous fringe; punctures mostly round, becoming slightly smaller posteriad; interstices smooth except those of last three tergites shallowly reticulated, larger to much larger than diameter of punctures except those on basal impressions of basal three abdominal tergites, which could be narrower than half the diameter of punctures.

**Figures 68–71.**
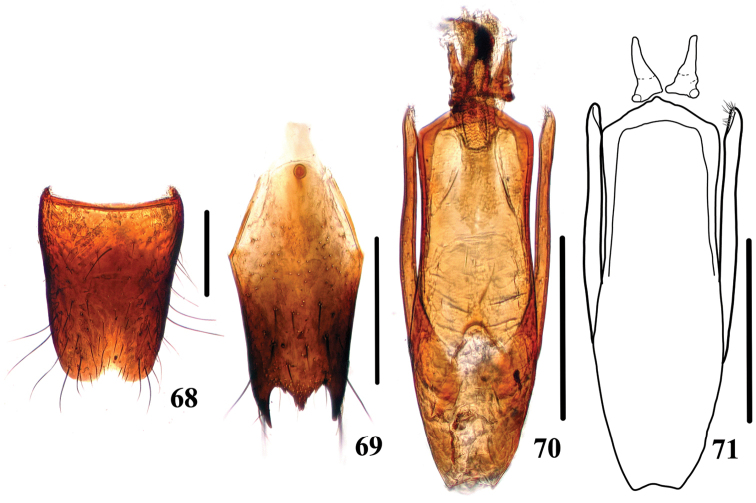
*Stenus tuyueyei*. **68** male sternite VIII **69** male sternite IX **70, 71** aedeagus. Scale bars: 0.25 mm.

Male. Sternite VIII (Fig. 68) with triangular emargination at middle of posterior margin; sternite IX (Fig. 69) with very long apicolateral projections, posterior margin with long and sharp median projection. Aedeagus (Figs 70, 71) robust; apical sclerotized area very wide and short with small and round tip; expulsion clasps large; parameres a little shorter than median lobe, slightly swollen in apical part, with 12 setae on apico-internal margins.

Female. Unknown.

#### Distribution.

China (Sichuan).

#### Remarks.

The new species can be readily separated from other related species by abdominal segment III fused on posterior half.

#### Etymology.

This species is named in honor of Mr. Yue-ye Tu who collected some specimens of the new species.

### Stenus dabashanus sp. n.

Taxon classificationAnimaliaColeopteraStaphylinidae

http://zoobank.org/66E26213-259C-4742-9E50-F0F57A822146

Figs 12 , 72–76

#### Type material.

**Holotype. China: Shaanxi**: ♂, glued on a card with labels as follows: “China: S-Shaanxi (Daba Shan), NW Pass 25km NW Zhenping, 32°01'N, 109°19'E, 2150 m, 11.VII.2001, M. Schülke leg. [C01-09].” “Holotype / *Stenus dabashanus* / Tang, Liu & Niu” [red handwritten label] (cSch). **Paratypes.** 1♂3♀♀, same data as for the holotype (1♀ in SHNU, rest in cSch); 1♀, Daba Shan creek vall., SE pass 20km NW Zhenping, 31°59'N, 109°22'E, 1680 m, 11.VII.2001, A. Smetana leg. (cSme); 1♀, Daba Shan creek vall., SE pass 25km NW Zhenping, 32°01'N, 109°19'E, 2150 m, 11.VII.2001, A. Smetana leg. (cSme).

#### Description.

Brachypterous; body reddish brown except interocular area of head and abdominal segments VIII–X blackish. Antennae, maxillary palpi and legs yellowish brown except antennal club infuscate.

BL: 2.9–3.8 mm, FL: 1.5–1.7 mm.

HW: 0.70–0.83 mm, PL: 0.50–0.60 mm, PW: 0.50–0.60 mm, EL: 0.50–0.63 mm, EW: 0.60–0.68 mm, SL: 0.31–0.38 mm.

Head 1.15–1.21 times as wide as elytra, interocular area with two deep longitudinal furrows, median portion convex, extending beneath the level of inner eye margins; punctures round, mostly well delimited, slightly larger and sparser on median area than those near inner margins of eyes, diameter of large punctures about as wide as basal cross section of antennal segment II; interstices faintly reticulated, much narrower than half the diameter of punctures except those along the midline of the convex median portion, which may be twice as wide as diameter of punctures. Paraglossa coniform.

Pronotum 0.96–1.05 times as long as wide; disk uneven, with median longitudinal furrow throughout; punctures confluent, of similar size to large punctures of head; interstices reticulated, narrower than half the diameter of punctures everywhere.

Elytra 0.83–0.93 times as long as wide, disk moderately uneven with distinct longitudinal humeral impression, distinct postero-lateral impression and long sutural impression, suture moderately convex; punctures longitudinally confluent and slightly larger than those of pronotum; interstices faintly reticulated, narrower than half the diameter of punctures.

Legs with hind tarsi 0.73 times as long as hind tibiae, tarsomeres IV deeply bilobed.

Abdomen cylindrical; line-like paratergites present only in segment III, tergites and sternites totally fused in segment IV–VI, tergite VII without apical membranous fringe; punctures distinctly smaller than those of elytra, gradually becoming smaller posteriad; interstices smooth, wider than the diameter of punctures except some on tergites III and IV, which could be smaller.

**Figures 72–76.**
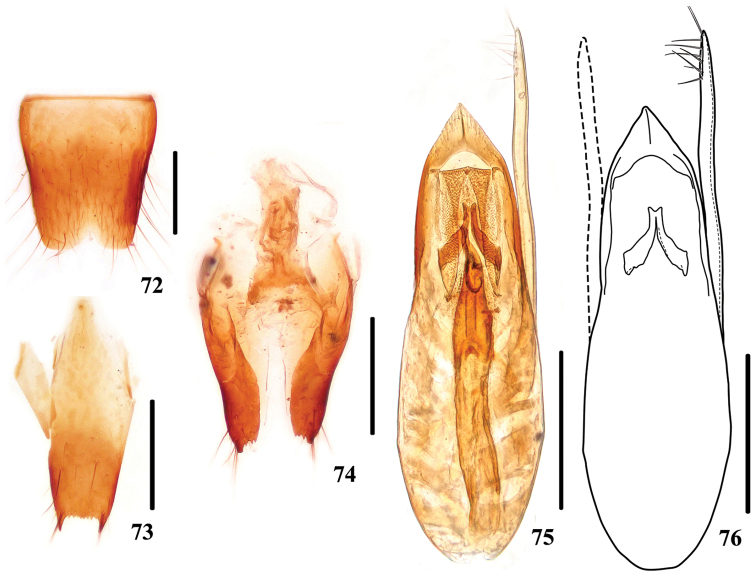
*Stenus dabashanus*. **72** male sternite VIII **73** male sternite IX **74** valvifers and spermatheca **75, 76** aedeagus. Scale bars: 0.25 mm.

Male. Sternite VII with week emargination at middle of posterior margin; sternite VIII (Fig. 72) with semi-circular emargination at middle of posterior margin; sternite IX (Fig. 73) with long apicolateral projections, posterior margin serrate. Aedeagus (Figs 75, 76) slender, apical sclerotized area triangular with a keel along the middle; expulsion clasps large, strongly sclerotized; parameres much longer than median lobe, with 9–10 setae on apico-internal margins.

Female. Sternite VIII slightly prominent at middle of posterior margin; spermatheca (Fig. 74) weekly sclerotized, basal porch large and spermathecal duct with multiple bends.

#### Distribution.

China (Shaanxi).

#### Remarks.

The species can be easily recognized by distinctly darkened abdominal tip and reticulated forebody.

#### Etymology.

The specific name is derived from the type locality of this species.

### Key to species of the *Stenus flammeus* group

**Table:** 

1	Abdominal segment III without distinct paratergites, rudimentary lateral border present only on the anterior half, tergite and sternite entirely fused on the posterior half. Habitus: Fig. 11; sexual characters: Figs 68–71	***Stenus tuyueyei***
–	Abdominal segment III with line-like paratergites, tergite and sternite clearly separated	**2**
2	Smaller with BL=2.9–3.9 mm and FL=1.5–1.9 mm	**3**
–	Larger with BL=4.0–5.7 mm and FL=2.0–2.4 mm	**10**
3	Last three abdominal segments distinctly darker than the basal segments; head, pronotum and elytra with interstices reticulated. Habitus: Fig. 12; sexual characters: Figs 72–76	***Stenus dabashanus***
–	Abdominal segments unicolor; head, pronotum and elytra with interstices smooth or rarely reticulated only at the bottom of longitudinal furrow of pronotum	**4**
4	Head much wider than elytra with HW/EW=1.25–1.31. Habitus: Fig. 1; sexual characters: Figs 13–17	***Stenus pengzhongi***
–	Head less wider than elytra with HW/EW=1.04–1.19	**5**
5	Punctation of entire body very dense, head with interstices along the midline narrower than diameter of punctures. Habitus: Fig. 5; sexual characters: Figs 36–40	***Stenus punctidorsus***
–	Punctation of entire body relatively sparse, head with some interstices along the midline distinctly wider than diameter of punctures	**6**
6	Elytra especially sutural impression with punctation longitudinally confluent and interstices very narrow and ridge-like	**7**
–	Elytra especially sutural impression with punctation less confluent and interstices broader	**8**
7	Robust, HW/EW=1.03–1.07, PL/PW=0.91–0.95 and EL/EW=0.82–0.90. Habitus: Fig. 2; sexual characters: Figs 18–23	***Stenus jindingianus***
–	Slender, HW/EW=1.11–1.17, PL/PW=0.95–1.02 and EL/EW=0.91–0.96. Habitus: Fig. 3; sexual characters: Figs 24–29	***Stenus corniculus***
8	Pronotum narrower with PW=0.49–0.50 mm, head much wider than elytra with HW/EW=1.15–1.21. Habitus: Fig. 10; sexual characters: Figs 63–67	***Stenus jiajinshanus***
–	Pronotum broader with PW=0.54–0.63 mm, head less wider than elytra with HW/EW=1.04–1.09	**9**
9	Aedeagus (Figs 56, 57) with small apical sclerotized area; expulsion clasps long. Habitus: Fig. 8	***Stenus xilingmontis***
–	Aedeagus (Figs 61, 62) with large apical sclerotized area; expulsion clasps short. Habitus: Fig. 9	***Stenus zhoudeyaoi***
10	Pronotum much shorter than width with PL/PW=0.90–0.96. Habitus: Fig. 6; sexual characters: Figs 41–46	***Stenus paraflammeus***
–	Pronotum at most slightly shorter than width with PL/PW=0.99–1.07	**11**
11	Elytra much shorter than width with EL/EW=0.84–0.92	**12**
–	Elytra at most slightly shorter than width with EL/EW=0.98–1.03	**13**
12	Posterior margin of male sternite IX with long median projection; spermatheca with very long basal porch. Habitus: Fig. 5 in Tang, Zhao & Puthz, 2008; sexual characters: Figs 25–29 in Tang, Zhao & Puthz, 2008	***Stenus flammeus***
–	Posterior margin of male sternite IX with short median projection; spermatheca with short basal porch. Habitus: Fig. 7; sexual characters: Figs 47–52	***Stenus pseudoflammeus***
13	Body coloration darker with head entirely black; punctation of pronotum very confluent; interstices narrowed into rugae. Habitus: Fig. 6 in Tang, Zhao & Puthz, 2008; sexual characters: Figs 30–34 in Tang, Zhao & Puthz, 2008	***Stenus bostrychus***
–	Body coloration lighter with head brown to dark brown; punctation of pronotum less confluent; interstices mostly not narrowed into rugae. Habitus: Fig. 4; sexual characters: Figs 30–35	***Stenus daicongchaoi***

## Supplementary Material

XML Treatment for Stenus flammeus

XML Treatment for Stenus bostrychus

XML Treatment for Stenus pengzhongi

XML Treatment for Stenus jindingianus

XML Treatment for Stenus corniculus

XML Treatment for Stenus daicongchaoi

XML Treatment for Stenus punctidorsus

XML Treatment for Stenus paraflammeus

XML Treatment for Stenus pseudoflammeus

XML Treatment for Stenus xilingmontis

XML Treatment for Stenus zhoudeyaoi

XML Treatment for Stenus jiajinshanus

XML Treatment for Stenus tuyueyei

XML Treatment for Stenus dabashanus
